# Comparison of T2 Weighted, Fat-Suppressed T2 Weighted, and Three-Dimensional (3D) Fast Imaging Employing Steady-State Acquisition (FIESTA-C) Sequences in the Temporomandibular Joint (TMJ) Evaluation

**DOI:** 10.1155/2021/6032559

**Published:** 2021-12-22

**Authors:** Secil Aksoy, Kaan Orhan

**Affiliations:** ^1^Near East University, Faculty of Dentistry, Department of Dentomaxillofacial Radiology, Mersin 10, Turkey; ^2^Ankara University, Faculty of Dentistry, Department of Dentomaxillofacial Radiology, Ankara, Turkey; ^3^Ankara University, Medical Design Application, Research Center (MEDITAM), Ankara, Turkey; ^4^Department of Dental and Maxillofacial Radiodiagnostics, Medical University of Lublin, Poland

## Abstract

**Aim:**

Osteonecrosis can affect the mandibular condyle, and bone marrow edema may be a precursor in osteonecrosis development in temporomandibular disorder (TMD) patients. Early detection of bone marrow changes is crucial for occurring osteonecrosis. The purpose of this study was to compare the diagnostic value of fast spin-echo T2 weighted (FSE-T2W), fat-suppressed T2W (FS-T2W), and three-dimensional (3D) fast imaging employing steady-state acquisition (FIESTA-C) MR sequences for early detection of bone marrow changes as well as TMJ soft tissue alterations.

**Methods:**

A total of 60 joints with TMD were included in this study using a 1.5T MR machine (Signa HDxt, GE, Milwaukee, USA) using a dual surface TMJ coil. Qualitatively, the images were interpreted by two observers for disk configuration, disk position, joint fluid, and bone marrow changes. Quantitatively, signal intensity ratios (SIR) in the TMJ condyle, retrodiscal tissue, disk, and muscle were also measured using all tested sequences. Kappa coefficients were calculated to assess both intra- and interobserver agreements for each image set. The SIR of each sequence was compared using a one-way ANOVA Bonferroni-Dunn test.

**Results:**

Overall intraobserver kappa coefficients ranged between 0.35 and 0.88 for joint fluid and between 0.22 and 0.82 for bone marrow changes diagnosis, suggesting high intraobserver agreement for FS-T2W and 3D FIESTA-C sequences than FSE T2W sequence (*p* < 0.05). 3D FIESTA-C showed higher agreement values for disk configuration and position detection than other sequences.

**Conclusions:**

3D FIESTA-C sequences can be used and incorporated into routine MRI protocols for obtaining high-resolution TMJ MR images due to the short acquisition time and 3D nature of the sequence. Additional studies should be done for dynamic TMJ imaging with this sequence.

## 1. Introduction

The term temporomandibular disorders (TMD) refer to several clinical problems which involve the muscles, TMJ, and associated structures, or both [1, 2]. TMD is the most common etiological factor of nondental pain in the orofacial region and is considered to be a subgroup of musculoskeletal disorders [3–5], and its prevalence is very variable and ranges between 16 and 68% in the literature [6, 7]. Disc displacements are the most commonly encountered disorder type among these groups [8]. However, there is a discrepancy between objective diagnosis and subjective patient-reported pain and disc displacement may be observed nearly in one-third of the asymptomatic volunteer [7].

Diagnostic imaging is essential for detecting congenital, developmental malformations neoplasia, fractures, dislocations, ankylosis and disc displacement, inflammatory disease, and arthritis of the TMJ [9]. Magnetic resonance imaging (MRI) is considered to be the gold standard for evaluating the TMJ structures including the articular disc, ligaments, and muscles with excellent spatial and contrast resolution [10–12]. Furthermore, MRI is the only imaging modality that provides direct visualization of bone marrow in vivo [13]. Unlike biopsy, which is bound to specific areas, MRI provides a more global picture of the bone marrow cellular composition [13] and clearly distinguishes fat from other tissues [14].

MRI examination of TMJ is usually performed using T1-weighted (T1W) and proton-density-weighted or T2-weighted (T2W) pulse sequences. While osseous and discal tissues are best delineated on T1 and proton density-weighted images, inflammation and joint effusions are mainly demonstrated on T2W images [12]. However, the TMJ is considerably smaller than other joints, before morphological changes occur, inflammatory changes of the joints are hard to detect with standard methods. For this instance, new sequences were started to apply especially for bone marrow and inflammatory changes of TMJ in different studies. Several studies used frequency-selective fat-suppressed (FS) T2W sequence and found it to be more sensitive than fast spin-echo (FSE) T2W images in detecting marrow alterations and stated to be used instead of FSE T2W images [15–17] while the others used several sequences such as half-Fourier acquisition single-shot turbo spin-echo (HASTE) [18–20], fast low-angle shot (FLASH) [21, 22], steady-state free precession (SSFP) (true fast imaging with steady-state precession (true FISP) [23], balanced fast field echo (bFFE), balanced turbo field echo (bTFE), and fast imaging employing steady-state acquisition sequence (FIESTA) [24] especially for dynamic imaging of TMJ.

3D FIESTA sequence is an ultrafast pulse sequence and used for imaging of small structures such as cranial nerves (trigeminal neuralgia) [25], internal auditory canal [26], middle ear, or joints [24] in the literature. 3D FIESTA and FIESTA-C combine 3D volumetric data acquisition with fluid-sensitive steady-state imaging which adds phase cycling to the excitation pulse to minimize the build-up of artifacts in the residual transverse magnetization [24–26].

To the authors' knowledge, a comparison of the 3D FIESTA-C sequence in the detection of TMD with that of conventional sequences has not been assessed. Thus, the purpose of this study was to compare the diagnostic value of FSE T2, FS T2W, and 3D FIESTA-C MR sequences for early detection of bone marrow changes as well as TMJ soft tissue alterations.

## 2. Materials and Methods

The study population consisted of 122 patients who were admitted to the Near East University, Faculty of Dentistry, Department of Dentomaxillofacial Radiology for TMD. This study was conducted following the guidelines of the Declaration of Helsinki and was approved by our institutional ethical committee with IRB approval number YDU/2-6. Before undergoing any radiographic, intraoral, or extraoral examinations, the patients provided informed consent. Collected data were only accessible to the researchers.

All patients were examined clinically by a calibrated clinician (SA) for TMJ disorders according to the Diagnostic Criteria for Temporomandibular Disorders for Clinical and Research Applications (DC/TMD) [27, 28]. Standardized examination procedures include a comprehensive physical examination of TMJ, medical history of the patient, tenderness to palpation of masticatory muscles, measurements of the maximum mouth opening and lateral movements, limitations of the mandibular movements, assessment of the existence of joint sounds, and pain. The final diagnosis was defined as the result of integrating findings from the clinical examination according to DC/TMD guidelines and radiological examinations (cone beam computed tomography (CBCT), panoramic radiography, and MRI) [27, 28].

After each examination, the patients underwent bilateral TMJ MRI. This study was initially based on TMJ MR images of 122 patients who suffered from pain and dysfunction of TMJ. Images of 62 patients, whose MRIs were not diagnostically suitable for evaluation due to motion artifacts, and those who were found to have a syndromic disease or history of trauma, were excluded from the study. Moreover, only the patients who had anterior disc displacement with reduction and without reduction were included; all other displacements, sideway-partial or posterior disc displacements were excluded from the study.

The final study group was constituted as 60 patients (44 females and 16 male patients). The mean age of females was 38.1 while for males 32.4 age range 20-55 years.

All images were examined by using a 1.5T MR scanner (Signa HDxt, GE, Milwaukee, USA) using a dual 3-inch surface TMJ coil. First, an axial scout section was used to determine the mandibular condyle position, and then, MRI was performed on a median oblique sagittal location that was perpendicular to the long axis of the mandibular condylar head. The MRI protocol included the 3D FIESTA-C, FSE T2W, and frequency selected FS T2W images with the following parameters: T2W images (TR = 2500‐3000, TE = 93‐102 milliseconds (ms), echo train length (ETL) = 10, 256 × 256 matrix, 3 mm slice thickness, number of excitations (NEX) = 2, acquisition time (TA) = 3 minutes (min) 20 seconds (sec), and field of view (FOV) = 120 mm). Frequency selected FS T2W images were acquired with TR = 2500, TE = 80 ms, ETL = 10, 256 × 256 matrix, 3 mm slice thickness, NEX = 2, TA = 4 min 35 sec, and FOV = 120 mm. FIESTA images were acquired with TR = 10.3, TE = 4.2 milliseconds, flip angle 60, bandwidth 41.7 kHz 384 × 256 matrix, 3 mm slice thickness, NEX = 2, TA = 1 min 50 sec, and FOV = 120 mm. Oblique sagittal images were acquired perpendicular to the long axis of the condyle in both opened and closed mouth positions for T2W and FS T2W images with a bite-block that was available in variable size. The patients were instructed to move their mouths continuous slow free open-close during the process of FIESTA-MRI. The cycles of open-close movements for all the patients were unlimited. To minimize magnetic susceptibility artifacts (due to ultrashort TE and wideband thickness), constructive interference in steady-state (CISS) was performed (FIESTA-C).

Two oral and maxillofacial radiologists with an average of 8 years of experience evaluated the images. All observers were kept blind concerning the clinical signs and symptoms of the patients. Before image assessment, a calibration was made between the observers. They received a set of guidelines and classification criteria for disc displacements with MRI images. All observers evaluated the images twice in a 1-month term.

**Figure 1:**
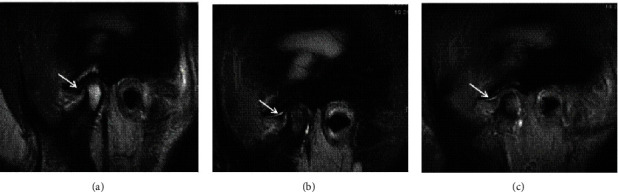
Sagittal MR images of the same patient using (a) FSE T2-W showing no effusion whereas (b) FS T2W and (c) 3D FIESTA-C show a moderate effusion in the superior joint compartment (arrows).

**Figure 2:**
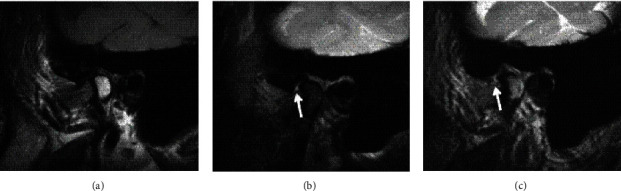
Sagittal MR images of another patient (a) FSE T2-W showing no effusion whereas (b) FS T2W and (c) 3D FIESTA-C show a mild effusion in the inferior joint compartment (arrows).

**Figure 3:**
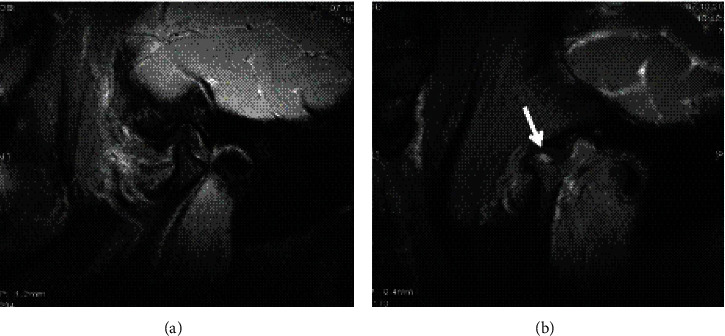
Bone marrow changes cannot be seen on (a) closed mouth-T2W images but be seen clearly on (b) open-mouth 3D FIESTA-C sequence of the same patient (arrows).

**Figure 4:**
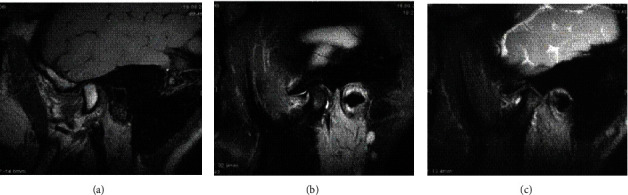
(a) FSE T2W image showing a normal bone marrow; (b, c) FS T2W and 3D FIESTA-C sequences of the same patient demonstrated decreased signal intensity within the mandibular condyle bone marrow.

The disc configuration was classified as biconcave, biconvex, and enlargement in the posterior band according to a previous study for this population [29]. The disc displacement of the TMJs was classified as normal, anterior disc displacement with reduction, anterior disc displacement without reduction again according to the same study [29]. Joint fluid was classified as no fluid, minimal, moderate, or marked fluid (Figures 1 and 2) while bone marrow changes were classified as normal bone marrow, edema, or osteonecrosis according to Larheim et al.'s studies [30, 31] (Figures 3 and 4).

**Figure 5:**
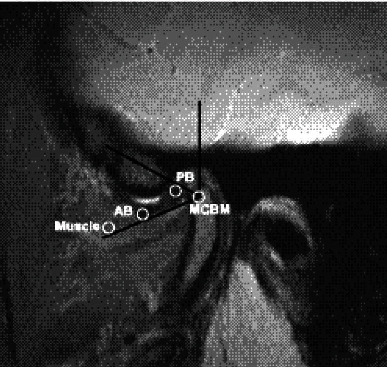
Selection of ROIs on MR images. AB: anterior band; PB: posterior band; MCBM: mandibular condyle bone marrow.

Quantitative analysis was also done in the study. There was a 3 mm diameter circular region of interest (ROI) in closed-mouth position placed in mandibular condyle bone marrow (MCBM) and disc (both from anterior band (AB) and posterior band (PB), and the muscle (lateral pterygoid muscle) (muscle)) on all MR sequences. Signal to noise ratio (SNR) is the highest at the center of the surface coil; thus, ROIs were placed as close as possible at the center of each tissue to diminish a partial volume effect. The signal intensity ratio (SIR) of each sequence and each tissue was measured. SIR was calculated as the ratio of the mean signal intensity in ROI and the standard deviation of the background noise, measured in a region of no signal (Figure 5).

The calculated SIR of each tissue compared a one-way ANOVA Bonferroni-Dunn test while kappa statistics were used to determine interobserver agreement. The kappa values were interpreted according to guidelines of Landis and Koch adapted by Altman [32]: *k* ≤ 0.20 poor, 0.21–0.40 fair, 0.41–0.60 moderate, 0.61–0.80 good, and 0.81–1.00 very good. The determination of significance level was done with the *t*-test using paired samples. Moreover, to assess intraobserver reliability, the Wilcoxon matched-pair signed-rank test was used for repeated evaluations. Results were considered significant at *p* < 0.05.

## 3. Results

### 3.1. Intraobserver Consistency

Repeated evaluation of MR images indicated no significant intraobserver difference for both observers (*p* > 0.05). Overall intraobserver consistency for observer 1 was rated at 92% and 95%, while the consistency for observer 2 was found at 89% and 91% between the two evaluations, respectively. All examinations were found to be highly reproducible for both observers, and no significant difference was obtained from the two evaluation sessions of the observers (*p* > 0.05).

### 3.2. Interobserver Consistency

Interobserver agreement on disc configuration, closed and open mouth disc position was moderate for T2W and FS T2W images whereas good agreement was found for 3D FIESTA-C sequences (*p* > 0.05). Statistical significance was found for disc position (open/closed), joint fluid, and bone marrow changes (*p* < 0.05). Interobserver agreement for joint fluid and bone marrow changes on FSE T2W images were fair while frequency-selective FS T2W and 3D FIESTA-C sequences showed very good agreement. The diagnosis was dramatically increased for joint fluid and bone marrow changes using frequency selective FS T2W and 3D FIESTA-C sequences which were statistically significant (*p* < 0.05) (Table 1).

Interobserver agreement for each variable showed that the highest kappa value for FSE T2W was anterior disc displacement without reduction and the lowest kappa value for joint fluid and bone marrow changes. Contrary to these results, FS T2W and 3D FIESTA-C images showed the highest kappa value for joint fluid and bone marrow changes which was statistically significant (*p* < 0.05) (Table 2). When joint fluid and bone marrow changes were examined in detail; the interobserver agreement of FSE T2W sequence was again fair whereas for FS T2W and 3D FIESTA-C images had a very good agreement (Table 3).

### 3.3. SIR Calculations

The mean SIRs of the MCBM, AB, PB, and muscle were calculated. The SIRs of all these issues on FS T2W and 3D FIESTA-C ratios were significantly higher than FSE T2W (*p* < 0.05). However, no significant difference was found between FS and 3D FIESTA-C sequences (*p* > 0.05) (Table 4).

## 4. Discussion

MRI is an excellent noninvasive imaging method for the evaluation of bone marrow and bone marrow alterations. Several reports have shown that bone marrow alterations were found to be associated with age, joint pain, internal derangement, osteoarthritis, and effusion [15, 33, 34].

It remains unclear whether bone marrow edema is a precursor for osteoarthritis or may occur separately from osteoarthritis [31, 34]. In a recent review study by Wahaj et al. [35], bone marrow edema, increased fluid level, and the pain were found to be associated with osteoarthritis in the majority of the TMJ studies. Most of the patients do not seek treatment until the late stage due to the early-stage osteoarthritis being asymptomatic; the need for an early detection method is necessary. Various MRI sequences (mainly T2W) were employed for the early detection of osteonecrosis and bone marrow changes. Joint pain and bone marrow alterations of the mandibular condyle result in higher signal intensity. Tanaka et al. [15] showed that FS T2W images are better than FSE T2W images for diagnosing the TMJ effusion. Similarly, the same results were reported for the detection of bone marrow edema by Morimoto et al. [16]. Also, TMJ pain is associated with high signal intensity in the posterior disc attachment (PDA) on FS T2W images [36]. Even though there was no significant correlation between pain score and signal intensity, the present correlation between the presence of TMJ-related pain and higher signal intensity was found by Kodama et al. [37] and the FLAIR sequence was recommended as a useful sequence in the clinical diagnosis of the painful TMJ. FLAIR sequence revealed a reduction in the signal intensity in TMJ with joint effusion [38]. A high signal intensity measurement of the disc and retrodiscal tissue was regarded as an objective sign of inflammation [39]. Orhan et al. [40] evaluated the signal intensity changes in the anterior and posterior band of the TMJ disc using MRI and reported that the increased SI in the posterior band significantly correlated with the progression of internal derangements while no significant difference was found in the anterior band. These differences may be explained by the higher volume density of the blood vessels and connective tissue content of the posterior band of the disc in TMD patients than the healthy individuals. In another study, Orhan et al. [17] revealed that SI values of the posterior band were significantly lower in the anemia patients than the healthy individuals whereas no statistically significant difference was found for the anterior band. In a recent study, a significant positive relationship between grading scores and SIR measurements for the posterior band of the disc and the retrodiscal tissue was found. SIR measurements considered as an objective measurement of inflammation seem to correlate with the detection of inflammation also during arthroscopy [41].

Few studies compared the diagnostic accuracy of the static proton density-weighted (PDW)/turbo spin-echo (TSE) T2W sequence dynamic HASTE sequence in internal derangements of TMJ. But they found that dynamic sequence is not a reliable alternative to static sequence [20] and static sequence remains as the gold standard; dynamic sequence provides additional information about the disc-condyle position [18].

Sun et al. [24] reported that additional information was obtained with the FIESTA sequence and this sequence could be applied in joints with clicking. Moreover, although FSE T2W imaging has a short acquisition time than FS T2W, it has still the limitation for motion artifact which can affect the image interpretation. In the present study, the acquisition time for fat-suppressed images (FS) was 4 min 35 seconds while the FSE T2W image acquisition time was 3 min 20 seconds. Therefore, new sequencing modalities and 3D imaging methods are still needed for TMJ imaging. For instance, Montesinos et al. [42] investigated the enhancement filters' effect on the image quality and visibility of disc, condyle, and articular eminence. They reported that different anatomical structures of the TMJ are better visualized with the application of the filter. Besides these filtering methods, new sequences that bring high-resolution imaging in small joints, hyaline cartilage, neoplasms, and peripheral nerves are being mandatory [43]. Chiba et al. [36] stated in their study that the nonuniform fat suppression in the suppressed T2W images reduces the reader's ability to evaluate the signal intensity of TMJ structures. Most previous studies used 1.5T MR imaging which influences T2W image interpretation because of this nonuniform fat suppression. Because of the wider chemical shift at high-field-strength MR imaging, the use of spectral fat suppression yields better results at 3T than at 1.5T. It should also be stated that the susceptibility artifacts at 3T are not strong enough to invalidate the advantage of a wider chemical shift. Therefore, at higher magnetic fields, better fat suppression may be achieved with a wider bandwidth in the fat frequency range and a shorter RF pulse [43, 44].

Recently, 3T imaging is also being used in TMJ [45–48]. Krohn et al. [45] investigated the potential of real-time MR imaging (dynamic TMJ imaging) using 3T and concluded the advantage of 3T for this kind of imaging. Yen et al. [46] developed an imaging protocol on a 3T system using the true FISP sequence that yielded an acceptable spatial and temporal resolution for dynamic MR imaging. A recent study used the T2 mapping technique to evaluate TMJ disc ultrastructure. They concluded T2 relaxation time measurements can enable an ultrastructural analysis of the articular disc of the TMJ [47].

Manoliu et al. [49] stated that the SNR at 3.0 T can be used to increase the spatial resolution when imaging the TMJ; thus, 3.0T should be preferred over 1.5T for imaging the TMJ.

Although this study was done in 1.5T, in the study, we aimed to employ a faster sequence obtained with the steady-state free precession (SSFP) method. These are gradient echo sequences that are shorter than the tissue's T1 and T2 time. RF pulses with a flip angle less than 90 degrees are sent to have short TR. Because of this, T2 weighting is increasing and images with high SNR are achieved. Due to fast imaging, movement and flow artifacts occur less than with other sequences. In the present study, the acquisition time 3D FIESTA-C sequence was less than 2 minutes which also allows the dynamic study of the TMJ. Based on the current results, the fat-suppressed with gradient echo sequences has significantly higher SIR and higher agreement for detection of joint fluid and bone marrow alterations.

It should be stated that radiologists should be aware of the advantages and disadvantages of the various fat suppression techniques available for 3T MR imaging to select the most appropriate technique for the evaluation of TMJ. Further studies should be done with various FS images such as STIR, CHESS, or Dixon techniques.

Moreover, Water excitation could conduct fat-water separation in various sequences which can be a potential to image small joints and cartilages including TMJ because of its relatively fast acquisition times and high SNR [43]. Based on the results of this current research, further studies on various FS and ultrashort TE sequences (such as 3D FIESTA-C sequences) can be recommended both with 1.5 and 3T for TMJ imaging with short acquisition time and decreased magnetic susceptibility artifacts.

The limitation of this study is that the imaging sections for dynamic MRI were planned on scout images in the axial plane. Moreover, because of the dynamic nature of FIESTA imaging during rapid jaw movement, MRI may still suffer from residual streaking artifacts due to the high degree of radial undersampling. The captured image velocity was limited to TMJ motion on dynamic imaging, in addition to the motion artifact that may occur during the dynamic imaging of TMJ. Further advances in image acquisition and iterative reconstruction techniques are needed to improve image quality and use of dynamic TMJ MR imaging.

## 5. Conclusion

In conclusion, 3D FIESTA-C sequences can be used and incorporated into routine MRI protocols for obtaining high-resolution dynamic TMJ MR images due to the short acquisition time and 3D nature of the sequence. Additional studies should be done for dynamic TMJ imaging with this sequence.

## Figures and Tables

**Table 1:** Kappa values for interobserver agreement according to MR sequences.

Variables	FSE T2		3D FIESTA-C		FS T2	
	Kappa	Standard error	Kappa	Standard error	Kappa	Standard error
Disc configuration	0.54	0.031	0.68	0.024	0.52	0.035
Disc position, closed mouth	0.48^1^	0.026	0.62^1^	0.038	0.58^1^	0.039
Disc position, open mouth	0.52^2^	0.036	0.69^2^	0.033	0.56^2^	0.027
Joint fluid	0.35^3^	0.028	0.88^3^	0.017	0.84^3^	0.019
Bone marrow changes	0.22^4^	0.044	0.82^4^	0.018	0.81^4^	0.010

Superscript numbers indicate statistical significance (*p* < 0.05).

**Table 2:** Comparison among MR sequences in terms of evaluated variables.

Variable	Category	FSE T2	FS T2	3D FIESTA-C
		Kappa		
Disc configuration	Biconcave	0.58	0.58	0.60
	Biconvex	0.52	0.54	0.61
	Enlargement of the posterior band	0.50	0.52	0.66
	Biplanar	0.48	0.54	0.62

Disc position, closed mouth	Superior	0.44	0.54	0.64
	Anterior	0.50	0.58	0.62

Disc position, open mouth	Superior	0.48	0.55	0.65
	ADD with reduction	0.48	0.54	0.62
	ADD without reduction	0.54	0.58	0.69

Joint fluid	No	0.58	0.80	0.80
	Minimal	0.24	0.84	0.83
	Moderate	0.26	0.88	0.85
	Marked	0.32	0.89	0.89

Bone marrow changes	Normal	0.18	0.83	0.82
	Edema	0.20	0.75	0.78
	Osteonecrosis	0.24	0.89	0.90

**Table 3:** The kappa values for joint fluids and bone marrow changes according to each observer.

Variables	FSE T2			
	Observer 1		Observer 2	
	Kappa	Standard error	Kappa	Standard error
Joint fluid	0.38^∗^	0.016	0.34^∗^	0.018
Bone marrow changes	0.21^∗^	0.024	0.29^∗^	0.026

3D FIESTA-C				
Joint fluid	0.86^∗^	0.017	0.88^∗^	0.029
Bone marrow changes	0.80^∗^	0.018	0.88^∗^	0.022

FS T2				
Joint fluid	0.86^∗^	0.019	0.84^∗^	0.025
Bone marrow changes	0.80^∗^	0.020	0.82^∗^	0.024

**Table 4:** Distribution of each tissue SIR (mean ± S.D.) according to MR sequences.

	T2-W^∗^	FS T2-W^∗^	3D FIESTA-C^∗^
MCBM	729.33 ± 102.18	895.74 ± 105.58	936.79 ± 109.07
AB	510.25 ± 62.01	703.5 ± 76.93	746.61 ± 76.92
PB	519.78 ± 64.81	754.36 ± 78.44	755.58 ± 79.72
Muscle	706.38 ± 99.60	872.12 ± 101.28	886.79 ± 107.02

MCBM: mandibular condyle bone marrow; AB: anterior band of the disc; PB: posterior band of the disc. ∗ indicates statistical significance.
